# Targeted Next-Generation Sequencing of a Deafness Gene Panel (MiamiOtoGenes) Analysis in Families Unsuitable for Linkage Analysis

**DOI:** 10.1155/2018/3103986

**Published:** 2018-01-15

**Authors:** Haiqiong Shang, Denise Yan, Naeimeh Tayebi, Kolsoum Saeidi, Afsaneh Sahebalzamani, Yong Feng, Susan Blanton, Xuezhong Liu

**Affiliations:** ^1^Department of Otolaryngology, University of Miami Miller School of Medicine, Miami, FL 33136, USA; ^2^Department of Otolaryngology-Head and Neck Surgery, Shandong Provincial Hospital Affiliated to Shandong University, Jinan 250021, China; ^3^Applied Physiology Research Center, Isfahan Cardiovascular Research Institute, Isfahan University of Medical Sciences, Isfahan, Iran; ^4^Neuroscience Research Center, Institute of Neuropharmacology, Kerman University of Medical Sciences, Kerman, Iran; ^5^Department of Medical Genetics, Kerman University of Medical Sciences, Kerman, Iran; ^6^Paediatric and Genetic Counselling Center, Kerman Welfare Organization, Kerman, Iran; ^7^Department of Otolaryngology, Xiangya Hospital, Central South University, Changsha, Hunan, China; ^8^John P. Hussman Institute for Human Genomics, University of Miami, Miami, FL 33136, USA

## Abstract

Hearing loss (HL) is a common sensory disorder in humans with high genetic heterogeneity. To date, over 145 loci have been identified to cause nonsyndromic deafness. Furthermore, there are countless families unsuitable for the conventional linkage analysis. In the present study, we used a custom capture panel (MiamiOtoGenes) to target sequence 180 deafness-associated genes in 5* GJB2* negative deaf probands with autosomal recessive nonsyndromic HL from Iran. In these 5 families, we detected one reported and six novel mutations in 5 different deafness autosomal recessive (DFNB) genes* (TRIOBP, LHFPL5, CDH23, PCDH15, *and* MYO7A)*. The custom capture panel in our study provided an efficient and comprehensive diagnosis for known deafness genes in small families.

## 1. Introduction

Hearing loss (HL) is one of the most common sensory disorders in humans. The prevalence of moderate, severe, and profound bilateral permanent hearing loss is estimated at 1 in 900 to 2500 newborns, with genetic causes accounting for 50% to 60% of cases [1, 2]. Among hereditary hearing impairments, approximately 70% are nonsyndromic, with deafness being the sole clinical feature [3]. The remaining 30% have HL, together with other associated clinical manifestations that constitute a syndrome. Over 145 distinct loci have been reported to be associated with nonsyndromic HL (NSHL), and there are more than 400 genetic syndromes that include HL (Hereditary Hearing loss Homepage, http:/hereditaryhearingloss.org/). Thus, it is critical to establish a genetic diagnosis of HL for the deaf person and their families.

For many decades, linkage analysis combined with the candidate genes approach has been the main tool to elucidate the genetics of HL. However, this approach is costly, time-consuming, and unsuitable for families with inadequate numbers of available affected individuals combined with locus heterogeneity. The advent of next-generation sequencing (NGS) technologies has the potential to overcome such limitations by making laboratories cost effective and simultaneously perform parallel sequencing of billions of nucleotides at once [4, 5]. Recently, NGS technologies have been adopted by a growing number of molecular genetic clinical laboratories. They offer the opportunity to identify variants in known disease genes, which is of critical importance to an extremely heterogeneous condition such as deafness [6]. To address the genetic heterogeneity of deafness more effectively and minimize the labor costs and expenses of conventional techniques, we developed a targeted genomic enrichment to capture and sequence all exons of 180 known and candidate deafness genes for nonsyndromic and syndromic forms of deafness [7]. In a proof-of-principle study, we demonstrated that the gene panel is sufficiently sensitive and specific for clinical deafness diagnostics [7, 8]. There have been also several other recent studies showing similar positive results using target enrichment approaches and massively parallel sequencing technologies [9, 10].

In the present study, we used this 180 targeted gene enrichment panel to detect genetic variants in 5 unrelated probands with a broad range of HL onset and severity. Of these, we identified 7 variants in 5 unrelated recessive families, including 1 reported mutation and 6 novel mutations in 5 different deafness autosomal recessive (DFNB) genes. Furthermore, the genotypes for these variants were consistent with the autosomal inheritance pattern of deafness in each family. The data suggested that our targeted gene panel provided a comprehensive and efficient genetic testing in the evaluation of deaf patients from families unsuitable for linkage analysis.

## 2. Materials and Methods

### 2.1. Families and Clinical Evaluation Subjects

5 unrelated families with recessive HL analyzed in the present study were recruited from Iran. The signed informed-consent forms were obtained from each participant or, in the case of a minor, from the parents. This study was approved by the local Institutional Review Board at the University of Miami (USA) and Isfahan University of Medical Sciences, Isfahan, Iran.

All clinical data of affected members in these families were obtained from questionnaire and standard audiometry according to current standards. Clinical evaluation included a thorough physical examination and otoscopy by a geneticist and an otolaryngologist. Pure-tone audiometry (PTA) or auditory brainstem response (ABR) was used to assess degree and progression of HL. The degree of HL was calculated on the average of the threshold at 0.25, 0.5, 1, 2, and 4 kHz. The following guideline was used to determine the degree of HL: normal hearing (≤25 dB), mild hearing loss (25~40 dB), moderate hearing loss (40~60 dB), severe hearing loss (60~80 dB), and profound hearing loss (>80 dB).

According to pedigree analysis and family information, we characterized hearing loss type as recessive (parents are normal hearing, possible consanguinity known, indicated by families R1 through R5). When possible, additional family members were also recruited for follow-up cosegregation analysis. Genomic DNA was extracted from peripheral blood or buccal cells using either the Gentra Puregene DNA isolation kit (Qiagen, Hilden, Germany) or the Puregene Buccal Cell Core kit (Qiagen, Hilden, Germany), respectively.

### 2.2. Target Enrichment Sequencing

We have developed a custom capture panel of 180 known and candidate genes associated with sensorineural hearing loss using the Agilent SureDesign online tool (https://earray.chem.agilent.com/suredesign/). A SureSelect custom kit (Agilent, Santa Clara, CA, https://www.agilent.com) with a target size of approximately 1.158 Mb encompassing 3494 regions was designed to cover genes associated with both syndromic and nonsyndromic hereditary HL. The target sequencing was processed at the Hussman Institute for Human Genomics (HIHG) Sequencing core, University of Miami. The Agilent's SureSelect Target Enrichment (Agilent, Santa Clara, CA) of coding exons and flanking intronic sequences for each exon by a solution based capture system was used according to the manufacturer's standard protocol [7]. Adapter sequences for the Illumina HiSeq2000 were ligated and the enriched DNA samples were prepared using the HiSeq2000 instrument (Illumina) following the standard methods. Average insert size was 180 bp and paired-end reads were produced.

### 2.3. Bioinformatics Analysis

Bioinformatics processing and data analysis were performed as previously described [7]. The Illumina CASAVA v1.8 pipeline was applied to assemble 99 bp sequence reads. The sequence reads were aligned using Burrows-Wheeler Aligner (BWA) to the human reference genome (hg19) [11] and variants were submitted using FreeBayes. An overall quality check of uploaded read data was then performed, including the coverage and average read depth of targeted regions and quality scores. Genesis 2.0 (https://www.genesis-app.com/) was used for variant filtering based on quality/score read depth and minor allele frequency (MAF) thresholds of 0.005 for recessive NSHL, as reported in the dbSNP141, the National Heart, Lung, and Blood Institute Exome Sequencing Project Exome Variant Server, Seattle, WA Project (Exome Variant Server, 2012), Exome Aggregation Consortium (ExAC) browser (http://exac.broadinstitute.org/). Variants meeting these criteria were further annotated according to specific standard terminology of American College of Medical Genetics and Genomics (ACMG) guidelines [12] that recommends classification of DNA variants into five categories including pathogenic, likely pathogenic, variant of uncertain significance (VUS), likely benign, and benign. To analyze the possible functional pathogenic effects of the missense variants, 2 types of prediction programs, SIFT, and Polyphen score were used. The Human Gene Mutation Database (http://www.hgmd.cf.ac.uk), the Deafness Variation Database (DVD) (deafnessvariationdatabase.org), and Clinvar (http://www.ncbi.nlm.nih.gov/clinvar/) were used as references to determine the novelty and probable pathogenicity of the allelic variations detected in our sequencing approach.

### 2.4. Sanger Sequencing

Candidate variations were confirmed via Sanger sequencing. Primer3, v. 0.4.0 (http://primer3.ut.ee), was used for primer design. PCR primers were summarized in Supplementary Table S1. Polymerase chain reaction (PCR) reactions were performed with 40–60 ng of genomic DNA and Taq DNA polymerase (Sigma) using standard protocols. PCR products were purified with Qiagen Qiaquick purification kit and bidirectionally sequenced using the ABI PRISM Big Dye Terminator Cycle Sequencing V3.1 Ready Reaction Kit and passed on the ABIPRISM 3730 DNA Analyzer (Applied Biosystems). DNA sequence analysis was performed with DNASTAR Lasergene software.

## 3. Results

### 3.1. Clinical Features of the 5 Families in Which Gene Variants Have Been Identified

DNA sequence variants were identified in 5 families segregating autosomal recessive HL. All affected members of the 5 families had severe to profound hearing impairment. With the exception of probands in R5 (VI:2 and VI:5) who suffer from Usher syndrome characterized by hearing loss, blindness due to retinitis pigmentosa, and vestibular dysfunction, none of them complained of syndromic disorders. Clinical information of the deaf probands was summarized in Table 1.

### 3.2. Variant Analysis

Targeted panel sequencing was performed on one affected member of each family. We first performed an overall quality check of uploaded read data, including the coverage and average read depth of targeted regions and quality scores. An average of 880 variants is obtained after filtering by gene features. Genesis 2.0 (https://www.genesis-app.com/) was then used for further variant filtering based on minor allele frequency [MAF thresholds of 0.005 for autosomal recessive nonsyndromic hearing loss (ARNSHL)] as reported in public databases and our internal database of > 5,000 individuals including Iranian samples. The subsequent conservation and pathogenicity scores analysis led to 2 to 5 predicted disease-causal variants [missense (~1.6%); splicing (~0.04%); stopgain (0.04%)] in each family for Sanger sequencing to study segregation. We detected 7 variants in 5 genes known to cause recessive hearing loss. We have classified 2 of the variants as uncertain based on the ACMG guidelines and because of the lack of data source for variant assessment.

### 3.3. Identification of Candidate Mutations of Hearing Loss in Each Family

**Figure 1:**
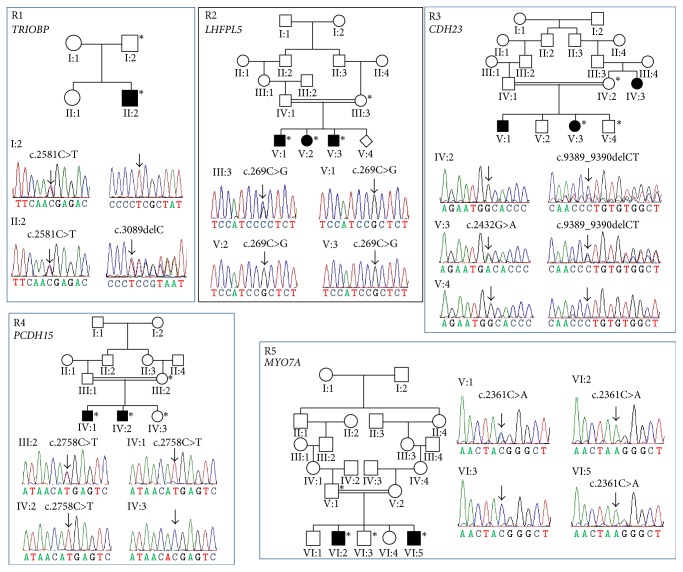
Pedigrees and sequence chromatograms of families with sequence variants and segregation indicating recessive inheritance (R1 through R5). Asterisk (*∗*) indicates DNA sample available. Filled squares or circles indicate affected individuals. Location of the variants is shown by an arrow.

The Iran community is an ancient population that is highly endogamous until now. In this ethnic group, we identified 3 homozygous and 4 compound heterozygous variants in 5 genes known to cause recessive hearing loss (Table 2). All the variants were found cosegregating with the deaf phenotype in extended family members; two compound heterozygous HL segregating mutations, c.2581C>T (p.R861X) and c.3089delC (p.P1030LfsX183) in* TRIOBP*, were detected in R1 family (Figure 1). Genotype confirmation using Sanger sequencing revealed that one patient (II-2) was heterozygous for these two mutations. A normal parent, I-1, was only heterozygous for p.R861X, consistent with the autosomal recessive inheritance pattern of the disorder.

A homozygous missense mutation c.269 C>G (p.P90R) in the* LHFPL5* gene was identified in the proband in R2, who showed bilateral congenital severe hearing loss. This novel missense mutation was located in an evolutionarily conserved domain. Subsequent Sanger sequencing confirmed the mutation in V:1 and revealed its presence in his two affected sisters in homozygous state and their unaffected mother was heterozygous for this variant. This further suggested that the p.P90R alteration in* LHFPL5 *gene would likely be considered a recessive pathogenic variant.

Two novel* CDH23* mutations including c.2432G>A (p.G811D) and c.9389_9390delCT (p.P3135RfsX19) were identified in individual V:3 of R3 family in a compound heterozygous state (Figure 1). Audiograms showed bilateral congenital profound hearing loss in both individuals V:1 and V:3. The presence of these variants in V:3 was confirmed by Sanger sequencing which also revealed compound heterozygosity for both of these nucleotide sequence changes in V:1. Genetic analysis of the two normal hearing parents revealed that IV:1 was only heterozygous for c.2432G>A (p.G811D) and IV:2 had only one abnormal allele [c.9389_9390delCT (p.P3135RfsX19)]. The two variants were absent from another unaffected individual V:4, which is consistent with the autosomal recessive inheritance pattern of the disorder.

We identified a homozygous hearing loss segregating variant c.2758 C>T (p.R920X) in the* PCDH15* gene in individual IV:1 of R4 family who has congenital profound hearing loss. This novel nonsense mutation (p.R920X) was located in the Cadherin repeat 8 of the PCDH15 protein. Sanger sequencing confirmed the mutation in a homozygous state in the proband IV:1 and his affected brother IV:2 was found homozygous for this mutation. An unaffected brother IV:3 was homozygous for the wild-type allele and the normal hearing mother III:2 was a heterozygous carrier of one mutant allele. All these observations favor an autosomal recessive mode of inheritance of the mutation.

Interestingly, the proband VI:2 in family R5 was diagnosed as syndromic hearing loss and referred to the clinics with a mild form of Usher syndrome. Individual VI:2 and his affected brother VI:5 had signs of retinitis pigmentosa at the time of diagnosis. They were found to carry a* MYO7A* homozygous nonsense mutation, c.2361C>A (p.Y787X). Their normal hearing brother was homozygous for the wild-type and the parent was heterozygous for the variant c.2361C>A (p.Y787X) in* MYO7A*. This is consistent with the autosomal recessive inheritance pattern of hearing loss.

We screened copy number variations (CNVs) as a part of the 180 deafness gene panel by investigating whether there were combined effects of the identified variants with CNVs in the same locus for the studied probands. There were no CNVs encompassing 2 or more exons gains nor losses found.

## 4. Discussion

The identification of pathogenic deafness-causing mutations was traditionally dependent on traditional method Sanger sequencing, which is expensive and time-consuming. In order to overcome this limitation, we applied a custom capture NGS panel, which combines target capture (TGE) and massively parallel sequencing (MPS) to rapidly sequence a set of 180 candidate genes associated with hearing loss. In the present study, the routine screening is initiated with* GJB2 *analysis because mutations in this gene have been identified worldwide in patients with hearing impairment. In the current study, we detected one reported and six novel mutations in 5 distinct deafness genes* (TRIOBP*,* LHFPL5*,* CDH23*,* PCDH15*, and* MYO7A)* in 5 recessive families. The nucleotide changes consist of two missense mutations, two frameshift indels mutations, and three nonsense mutations.

In family R1, one reported nonsense mutation c.2581C>T (p.R861X) and a novel frameshift mutation c.3089delC (p.P1030LfsX183) in the* TRIOBP* gene were found.* TRIOBP* encodes a filamentous-actin-binding protein that has been identified as the gene for DFNB28 deafness [13]. To date, at least 26 mutations have been reported in the* TRIOBP* gene in different populations. Nearly all of the previously reported mutations of* TRIOBP* causing HL are located in exon 6 [14]. However, the two compound heterozygous mutations of* TRIOBP* reported in the present study are observed in exon 7, suggesting that these regions of* TRIOBP* may also be the targets of mutations in HL. The* LHFPL5* gene encodes tetraspan membrane protein of hair cell stereocilia, which regulates transducer channel conductance and is required for fast channel adaptation [15]. Mutations in the* LHFPL5* gene have been reported in patients with nonsyndromic sensorineural deafness autosomal recessive type 67* (DFNB67)* [16]. To date, only few mutations in* LHFPL5 *have been linked to ARNSHL. The novel missense variant (c.269C>G; p.Pro90Arg) that we have identified in homozygous state in R2 family is located in the extracellular domain of the tetraspan membrane protein. The* CDH23* gene encodes a calcium-dependent cell-cell adhesion glycoprotein, which is responsible for stereocilia organization and hair bundle formation [17]. Mutations in the* CDH23* gene have been reported in patients with Usher syndrome 1D* (USH1D)* and nonsyndromic sensorineural deafness autosomal recessive type 12* (DFNB12)* [18]. In this study, we identified two novel compound heterozygous mutations including a missense variant (c.2432G>A; p.G811D) and a frameshift deleterious mutation (c.9389_9390delCT; p.P3135RX19) in the* CDH23* gene. These two mutations were located in exon 22 and exon 65 of the* CDH23* gene, respectively. As the affected members in R3 family had only hearing loss without other syndromes, the two novel mutations might be related to* DFNB12*.

In R4 family, a novel nonsense mutation caused by c.2758 C>T (p.R920X) was detected in* PCDH15*, which is responsible for both Usher syndrome 1F* (USH1F)* and deafness autosomal recessive type 23* (DFNB23)* hearing loss [19]. The gene encodes Protocadherin 15, which is expressed in the neurosensory epithelium of the ear [20, 21]. The nonsense mutation (p.R920X) associated with nonsyndromic deafness is located in the Cadherin 8 of Extracellular (EC) domain. We hypothesize that this nonsense mutation impaired the ability of the EC domain to interact with each other to form the lateral links, hence destabilizing stereocilia bundles and causing deafness. The* MYO7A* gene encodes the actin-binding motor protein Myosin VIIA protein, which is involved in differentiation, morphogenesis, and organization of cochlear hair cell bundles. Mutations in the* MYO7A* gene have been identified to be associated with nonsyndromic hearing loss* (DFNB2*,* DFNA11)* and Usher Syndrome type 1B* (USH1B)* [22–24]. The novel nonsense mutation (p.Y776X) identified in this study is located in the second isoleucine-glutamine (IQ) motif domain of the Myosin VIIA protein. IQ motifs are calmodulin- (CaM-) binding domains that form the neck of unconventional myosin proteins. The number of IQ motifs determines the step size and the velocity of the motor molecule [25]. We speculate that this nonsense mutation p.Y776X would reduce the number of IQ motifs to impair the motor function. Therefore, it is expected that the mutation could cause an inherited form of hearing loss.

Of the five Iran families enrolled in this study, seven mutations including three homozygous and four compound heterozygous mutations were identified. The homozygous mutations were commonly found in this community, and the reason is that Iran is still a highly endogamous population. Our successful identification of pathogenic mutations will provide genetic analysis for the clinical diagnosis of hearing loss.

## 5. Conclusion

In this study, we investigated the genetic epidemiology of hereditary hearing loss using target capture and massively parallel sequencing in families not suitable for linkage mapping. Totally, 7 variants in 5 different genes were identified in 5 unrelated* GJB2* negative families, suggesting the usefulness of this targeted massively parallel sequencing for comprehensive testing for all known deafness genes. Our successful identification of several pathogenic mutations indicates that gene targeted sequencing is a highly effective and powerful tool for clinical and population genetic studies of heterogeneous disorders.

## Figures and Tables

**Table 1:** Clinical manifestation of the patient cohort with recessive hearing loss.

Family ID	Individual ID	Gender	Age of onset (year)	Mean hearing threshold (dB)		Shape of audiogram	Severity
				Left	Right		
R1	II:2	Female	Birth	51	41	High-frequency gently sloping	Moderate
R2	V:1	Male	Birth	74	74	Flat	Moderate
	V:2	Male	Birth	106	100	Flat	Profound
	V:3	Male	Birth	108	104	Flat	Profound
R3	V:3	Female	Birth	106	105	Flat	Profound
R4	IV:1	Male	Birth	112	109	Flat	Profound
	IV:2	Male	Birth	105	104	Flat	Profound
R5	VI:2	Male	Birth	114	114	Flat	Profound
	VI:5	Male	Birth	104	42	Flat	Profound

**Table 2:** Mutations identified in the cohort of 5 families.

Family ID	Gene	DFN locus	Chromosome	Exon	Nucleotide change	Amino acid change	Zygosity	Pathogenic	Polyphen2	SIFT	Novel or HGMD
R1	*TRIOBP*	DFNB28	22	7	c.2581C>T	p.R861^*∗*^	Heterozygous	Pathogenic	—	—	Reported
	*TRIOBP*	DFNB28	22	7	c.3089delC	p.P1030Lfs^*∗*^183	Heterozygous	Pathogenic	—	—	Novel
R2	*LHFPL5*	DFNB67	6	1	c.269 C>G	p.P90R	Homozygous	Uncertain significance	1	0	Novel
R3	*CDH23*	DFNB12	10	22	c.2432G>A	p.G811D	Heterozygous	Uncertain significance	1	—	Novel
	*CDH23*	DFNB12	10	65	c.9389_9390delCT	p.P3130Rfs^*∗*^19	Heterozygous	Pathogenic	—	—	Novel
R4	*PCDH15*	DFNB23	10	23	c.2758 C>T	p. R920^*∗*^	Homozygous	Pathogenic	—	—	Novel
R5	*MYO7A*	USH1B	11	20	c.2361C>A	p. Y787^*∗*^	Homozygous	Pathogenic	—	—	Novel

## Supplementary Materials

Supplementary Materials Table S1. PCR primers surrounding the variants of 5 genes used in the experiments.
