# Supplementary material for: Targeted Next-Generation Sequencing of a Deafness Gene Panel (MiamiOtoGenes) Analysis in Families Unsuitable for Linkage Analysis
Source: Biomed Res Int. 2018 Jan 15;2018:3103986. doi: 10.1155/2018/3103986 (PMC5820677; doi:10.1155/2018/3103986)
Supplement: Supplementary Materials — Table S1. PCR primers surrounding the variants of 5 genes used in the experiments. [file 3103986.f1.doc]

Table S1. PCR primers surrounding the variants of 5 genes used in the experiments.

| **Gene** | **Forward sequence** | **Reverse sequence** |
| --- | --- | --- |
| TRIOBP | CCTGTACCCGACAGGACAAT | GGGTTATGGGAGGAAGAGGT |
| TRIOBP | GGCCAACTCAGAGTGATGGT | CCCAATACACACAGGTGCAG |
| LHFPL5 | caccaaaggcaatccagact | gggatgttagctggagcaga |
| CDH23 | tacagactccccagccattc | aggaagaaggcaagtggtga |
| CDH23 | caccaaaggcaatccagact | gggatgttagctggagcaga |
| PCDH15 | tgatctggctacatttcagctc | tccaagatgtgagataccaagtg |
| MYO7A | gcagggcaggcattattcta | ccccaaaaacactccagaag |
|  |  |  |
|  |  |  |
|  |  |  |
|  |  |  |
|  |  |  |
|  |  |  |
|  |  |  |
|  |  |  |
